# Long-Term Quality of Life and Pregnancy Outcomes of Differentiated Thyroid Cancer Survivors Treated by Total Thyroidectomy and I^131^ during Adolescence and Young Adulthood

**DOI:** 10.1155/2016/7586482

**Published:** 2016-02-08

**Authors:** Melanie Metallo, Lelia Groza, Laurent Brunaud, Marc Klein, Georges Weryha, Eva Feigerlova

**Affiliations:** ^1^Department of Endocrinology, University Hospital Center of Nancy, 54000 Nancy, France; ^2^Department of General and Endocrine Surgery, University Hospital Center of Nancy, 54000 Nancy, France; ^3^Department of Endocrinology, University Hospital Center of Poitiers, 86000 Poitiers, France

## Abstract

*Introduction*. Differentiated thyroid cancer (DTC) is rare and confers good prognosis. Long-term health related quality of life (HRQoL) and pregnancy outcomes are not well known in subjects treated during adolescence and young adulthood.* Methods*. Cross-sectional analysis of HRQoL and global self-esteem, using SF-36 and ISP-25 surveys, and of pregnancy outcomes in female survivors of DTC treated by total thyroidectomy and I^131^ before age of 25 years.* Results*. Forty-five of 61 patients (74%) responded to the survey. Cumulative I^131^ activity was ≤3.85 GBq in 18 subjects and >3.85 GBq in 27 subjects. Mean time from diagnosis was 7.6 ± 5.2 years for the group ≤ 3.85 GBq versus 16.9 ± 11.6 years for the group > 3.85 GBq (*P* < 0.05). No significant alteration in long-term HRQoL and global self-esteem was observed. Thirty pregnancies after I^131^ were noted in patients from the group > 3.85 GBq and 10 in patients from the group ≤ 3.85 GBq. Frequency of miscarriages was of 17% (group > 3.85 GBq) and 10% (group ≤ 3.85 GBq) with 9 and 24 live births, respectively. No congenital malformations or first year mortality was noted.* Conclusion*. Long-term HRQoL, global self-esteem, and pregnancy outcomes are not affected in young female survivors of DTC.

## 1. Introduction

Differentiated thyroid cancer (DTC) is rare and its incidence in Europe in 2012 has been estimated as 6.3 per 100 000 with a mortality rate of 0.4 per 100 000 [1]. In spite of its relatively good prognosis, relapsing disease is frequent especially in young patients and long-term follow-up is warranted [2–4]. This may produce emotional, psychosocial, or physical limitations and implies the necessity for measurement of health related quality of life (HRQoL) in order to identify patients in need [5, 6]. One of the generic instruments largely used in this context is the generic SF-36 health questionnaire evaluating HRQoL in eight domains [7] which has previously been used in patients with thyroid cancer [8–11].

Up till now, existing studies report short-term HRQoL of patients with DTC [8, 10, 12]. However, data on a long-term HRQoL are rare [10]. The Swedish SF-36 survey has shown no alteration of mental and physical quality of life in 77 patients who underwent total thyroidectomy and RAI for DTC [10]. Recently, a national wide Swedish study has evaluated long-term HRQoL in 279 DTC survivors registered in the national population-based Swedish Cancer Registry [11]. Results of this survey demonstrate that half of the study participants were concerned, 15 years after initial diagnosis, of experiencing a disease recurrence and had a significantly lower HRQoL compared to subjects without concerns [11]. To our knowledge, a long-term HRQoL of survivors of DTC diagnosed and treated during adolescence and young adult age has not been reported.

One of the treatment options of DTC is radioiodine ablation (RAI), which can produce long-term consequences, especially on fertility and pregnancy outcomes [3, 4, 13]. This may represent an additional burden for patients in reproductive age. Although limited by small sample sizes and select population sampling, the literature data suggest that there is no significant impact of RAI on pregnancy outcomes [14–16]. Balenović et al. [17] reported outcomes of 49 pregnancies in the population of 76 women treated by therapeutic dose of RAI in adulthood with a median activity of I^131^ prior to pregnancy of 3.7 GBq, with range of 2.96–28.86 GBq. Thirty-five children (72%), 5 (10%) miscarriages, and 9 (18%) induced abortions were noted. There were no congenital malformation and first year mortality. A higher therapeutic dose (>3.7 GBq) did not have a significant impact on pregnancy outcomes. Similarly, in the largest French study [15], which included 206 pregnancies, no significant impact on pregnancy outcomes has been demonstrated. However, no such question has been addressed specifically to young patients.

Altogether, literature data indicate insufficient information concerning long-term HRQoL and pregnancy outcomes in patients with DTC diagnosed and treated at young age. We therefore conducted a cross-sectional study aiming at evaluating a long-term quality of life and global self-esteem of female survivors of DTC diagnosed and treated by total thyroidectomy and I^131^ during adolescence and young adulthood. We also assessed pregnancy outcomes after total thyroidectomy and treatment by I^131^.

## 2. Methods

### 2.1. Participants

The University Hospital in Nancy is a tertiary referral centre in the region of Lorraine (France) for treatment of thyroid cancer and keeps a register of patients, available from 2004, based on a diagnostic code. Females who were followed in the Department of Endocrinology of the University Hospital in Nancy between 1 January 2004 and 31 December 2013 for histologically proven differentiated thyroid cancer (DTC) and who were below 25 years of age at the time when they were first treated for DTC were eligible to participate in this retrospective study.

Based on medical records, a telephone call was made to patients who fulfilled appropriate inclusion and exclusion criteria to introduce the study and to solicit participation. Letters were subsequently sent to those patients who were willing to participate with information about the research project along with a consent form. Inclusion criteria were as follows: age ≤ 25 years at the time of the first treatment for DTC. Patients aged above 25 years at diagnosis of DTC and those presenting with undifferentiated thyroid cancer or medullary thyroid cancer were excluded from the study.

A total of 90 patients eligible to participate were retrieved from the database. Twenty-eight subjects could not be contacted because of the missing data (incomplete address) in the medical records and 1 patient refused to participate. The questionnaires were sent via mail to 61 potential participants. Written informed consent was obtained from all patients prior to inclusion.

All patients underwent total thyroidectomy followed by radioactive iodine (RAI) ablation by I^131^ after thyroid hormone withdrawal or after rhTSH stimulation at dose ranging from 1.48 to 3.7 GBq (40–100 mCi) as initial treatment. All patients were subsequently under TSH-suppressive treatment and were advised to avoid pregnancy after each I^131^ administration for 6 months. Due to modifications of practice guidelines over the study period between 1974 and 2013 the follow-up was not standardized. All patients diagnosed with a disease recurrence defined as increase in serum thyroglobulin and detection of iodine avidity on whole body diagnostic I^131^ scan (0.11–0.15 GBq, 3-4 mCi), or positive finding on neck ultrasonography, computed tomography, or fusion 2-deoxy-2-[18F]fluoro-D-glucose whole body positron emission tomography/computed tomography (18F FDG-PET/CT), received 3.7 GBq (100 mCi) of I^131^. TNM staging was performed according to the criteria of the American Joint Committee on Cancer 2009.

### 2.2. Health Related Quality of Life Assessment

Health related quality of life was evaluated using the self-administered Short Form-36 version 2 (SF-36 v2) [7, 19]. The questionnaire covers 8 health domains (functioning, physical and social domain, physical and emotional domain, mental health, pain index, vitality, general health perceptions, and health transitions) [7] and has previously been used in patients with thyroid cancer [8, 9]. To assess dimensions of global self-esteem, a shortened version of Physical Self-Inventory [18] consisting of 6 single domains (global self-esteem, physical self-worth, sport competence, physical condition, attractive body, and physical strength) was used. The results of SF-36 and ISP-25 were compared with healthy subjects from the French reference population [18, 19].

### 2.3. Reproductive and Fertility Outcome Assessment

To evaluate fertility and reproductive outcomes a four-part self-administered instrument was developed by investigators after consideration of the relevant data available in the literature. The questionnaire was sent to participants by mail and took approximately 30 to 45 minutes to complete. All responses were kept confidential. The survey addressed the following domains. The first domain provided information on the self and familial medical history, treatment, tobacco and alcohol consumption of responders, weight, and size (11 items). The second domain concerned demographic data (5 items). The third domain of the questionnaire focused on gynaecological history (12 items). The fourth domain recorded data concerning reproductive and fertility outcomes (miscarriage, abortion, prematurity, low birth weight, birth defects, and assisted reproductive treatment, 8 items). Subjects were invited to add free comments at the end of the questionnaire.

## 3. Results

### 3.1. Characteristics of Study Participants

**Figure 1:**
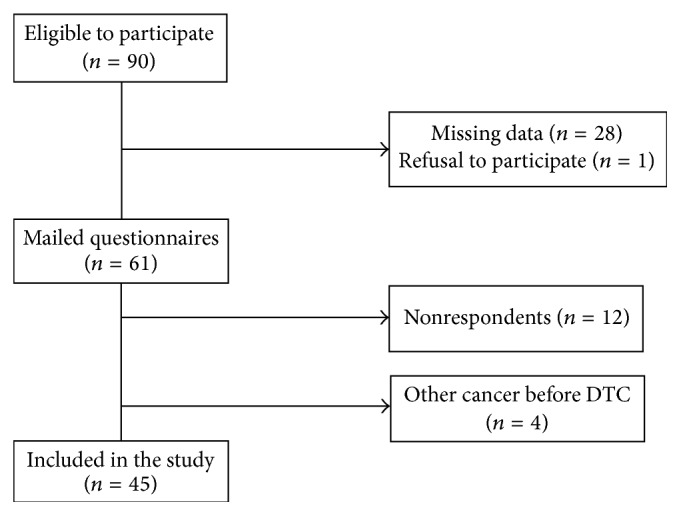
Flow chart of study participants.

Of 90 subjects who were eligible to participate, 61 received questionnaire between December 2014 and June 2015. Twenty-eight subjects could not be contacted because of the missing data (incomplete address) in the medical records and 1 patient refused to participate. Four females presented a history of another malignancy preceding diagnosis of thyroid cancer (acute lymphoblastic leukemia, *n* = 3, and Ewing sarcoma, *n* = 1) and were excluded from the study. Finally, out of 61 subjects who received the questionnaire, 45 returned the questionnaire, for a survey response rate of 74%. Flow chart of study participants is detailed in Figure 1.

The patients were divided into two groups according to the cumulative dose of I^131^: first, lower dose (≤3.85 GBq) and second, higher dose (>3.85 GBq). The mean age at diagnosis of DTC was similar for both groups. Compared to the patients from the lower dose group, the patients from the higher dose group had a significantly longer mean follow-up period, 7.6 ± 5.2 versus 16.9 ± 11.6 years, respectively (*P* < 0.05). Four patients presented with pulmonary metastases and 1 patient presented with bone metastases at diagnosis. Mean cumulative dose of I^131^ was 3.5 versus 8.3 GBq for the first and second group, respectively. Total dose was unknown for 6 females from the second group, but it was superior to 3.85 GBq. Characteristics of the participants are detailed in Table 1.

### 3.2. Quality of Life and Global Self-Esteem

**Figure 2:**
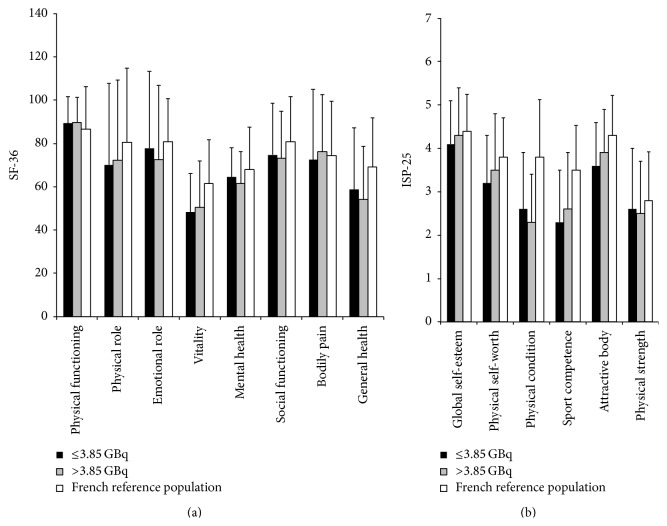
Results of SF-36 (a) and ISP-25 (b) for the two groups of patients (cumulative I^131^ activity ≤ 3.85 GBq versus cumulative I^131^ activity > 3.85 GBq) and the French reference population [18, 19]. Scores for each questionnaire component are represented as means (±SD).

The components of the SF-36 and ISP-25 questionnaires are detailed in Table 2. A total of 42/45 subjects (93%) responded to the SF-36 and 40/45 (89%) to the ISP-25 questionnaires. The SF-36 and ISP-25 results were compared with the corresponding data from a French reference population [18, 19]. No significant difference between the cancer group and the healthy French population was observed. There was a nonsignificant decrease in physical condition parameter in both cancer groups compared to the French healthy population [18, 19]. No differences between the different parameters in the higher dose cancer group and the lower dose cancer group were observed (Figure 2).

### 3.3. Pregnancy Outcomes

Mean cumulative dose of I^131^ administered prior to pregnancy was 3.74 GBq for the first group and 7.88 GBq for the second group (Table 3). Patients' ages at the time of pregnancy were in mean 26.3 and 27.3 years for the lower and the higher dose groups, respectively. The mean interval between the last administration of I^131^ and pregnancy was comparable for both study groups and was 69.7 months for the patients who received cumulative I^131^ dose ≤ 3.85 GBq and 75.3 for the patients with cumulative I^131^ dose > 3.85 GBq. Forty pregnancies after administration of I^131^ were observed: 30 in patients who received cumulative dose of I^131^ > 3.85 GBq and 10 in patients who received a dose of ≤3.85 GBq. Frequency of miscarriages was of 17% in females from the group > 3.85 GBq and 10% in patients with cumulative I^131^ dose ≤ 3.85 GBq. In total, 9 and 24 live births were noted for the first and the second groups, respectively. The data are detailed in Table 3. No birth defects or first year mortality was observed. All children were in good health condition at the time of study.

## 4. Discussion

Herein, we evaluated a long-term health related quality of life and pregnancy outcomes in young female thyroid cancer survivors from the region of Lorraine in France. Our study highlights that long-term quality of life and global self-esteem are not affected. Up till now, little has been published on a long-term HRQoL in patients with DTC. The Swedish SF-36 survey has shown no alteration of mental and physical quality of life in 77 patients of all ages who underwent total thyroidectomy and RAI for DTC [10]. Recently, a national wide Swedish study including 279 DTC survivors (218 females of mean age of 51 years) demonstrated a significantly poorer HRQoL in nearly 50% of the study participants, even 15 years after initial diagnosis of DTC [11]. Our study is the first study to assess HRQoL in patients diagnosed and treated by total thyroidectomy and I^131^ in adolescence and young age. The high response rate (74%) to our survey reflects interest of subjects and ongoing health concern despite having completed the cancer therapy. Interestingly, information-seeking behavior in cancer survivors and desire to receive additional health related information has been observed [20]. Thus, implementation of management strategies to better meet the patients' needs after completion of cancer therapy has recently been suggested [20–22]. Moreover, in spite of excellent prognosis of DTC, HRQoL of DTC survivors may be affected by life long thyroid hormone supplementation and in some cases by disorders of calcium homeostasis [23].

In terms of pregnancy outcomes, to our knowledge, the here presented study is the first to be addressed specifically to the population of long-term DTC survivors diagnosed and treated during adolescence or young adult age. Forty pregnancies after administration of I^131^ were observed: 30 in patients who received cumulative dose of I^131^ > 3.85 GBq and 10 in patients who received ≤3.85 GBq. Frequency of miscarriages was of 17% in females from the group > 3.85 GBq and 10% in patients with cumulative I^131^ dose ≤ 3.85 GBq. Our observations are consistent with the data for the French general population [24] and with the data reported for the French population with DTC diagnosed and treated in adulthood (18% miscarriages out of 152 pregnancies after thyroidectomy and cumulative I^131^ activity < 0.37 GBq and 21% miscarriages out of 178 pregnancies and cumulative I^131^ activity ≥ 3.70 GBq) [25]. Based on the statement of the French National Authority for Health (http://www.has-sante.fr/portail/upload/docs/application/pdf/2010-07/ald_30_gm_cancer__thyroide_web.pdf) avoiding pregnancy for 6 months after I^131^ treatment is suggested. The effects of other factors such as thyroid hormone status on the pregnancy outcomes were not investigated and this topic is out of the scope of this paper.

The conclusions of this study should be applied with caution since its sample population is not truly a reflection of the total French population of female patients diagnosed with DTC at adolescent age and young adulthood. However, the high response rate to this study represents less serious potential for a nonresponse bias. Furthermore, majority of the study participants were still in their reproductive age and report their real-life experience.

Altogether our results suggest that quality of life of young female thyroid cancer survivors is not affected. The results allowed us to evaluate the quality of life over a longer time period and incite new questions regarding information strategies for patients who underwent active thyroid cancer treatment especially at young age.

## Figures and Tables

**Table 1:** Clinical and demographic characteristics of patients diagnosed and treated with differentiated thyroid cancer before age of 25 years.

	Cumulative dose of I^131^	
	≤3.85 GBq	>3.85 GBq
Number	18	27
Diagnosis before age of 18 years (yes)	5 (28%)	12 (44%)
Age at diagnosis (years)	19.9 ± 3	19.3 ± 3.9
Actual age (years)	27.7 ± 6.7^*∗*^	36.1 ± 11.1^*∗*^
Time since diagnosis (years)	7.6 ± 5.2^*∗*^	16.9 ± 11.6^*∗*^
Histology		
Papillary	16	25
Follicular	2	2
TNM stage		
T?	0	4
T1	7	3
T2	10	10
T3	1	6
T4	0	4
N0	15	8
N1	3	19
M0	18	23
M1	0	4
Administration of I^131^		
Age at first treatment	19.9 ± 3	19.3 ± 3.9
Cumulative dose	3.54 ± 0.47^*∗∗*^	8.25 ± 1.64^*∗∗*^
Number of administration times	0.96 ± 0.2^*∗∗*^	2.7 ± 1.7^*∗∗*^
Marital status		
Single	10	6
Engaged	5	8
Married	3	11
Divorced	0	1
Missing	0	1
Education level		
Without diploma	1	1
Professional diploma	1	9
“Bac-level” diploma	9	10
University studies	12	15
Missing	0	1
Profession		
Student	6	3
Professional activity (yes)	8	21
Smoking		
No	11	6
Active	6	1
Stopped	1	20
IMC (kg/m^2^)	25.9 ± 4	23.7 ± 5

Data are presented as mean (±SD) or number (percentage).

^*∗*^ *P* < 0.05; ^*∗∗*^ *P* < 0.001.

**Table 2:** Long-term quality of life and global self-esteem of patients diagnosed with differentiated thyroid cancer before age of 25 years.

	Cumulative dose of I^131^						*P*
	≤3.85 GBq			>3.85 GBq			
	*n*/*N*	Mean	SD	*n*/*N*	Mean	SD	
SF-36							
Physical functioning	16/18	89.6	10.7	26/27	88.7	11.6	*0.841*
Physical role	16/18	73.2	37.2	26/27	69.2	37.3	*0.967*
Emotional role	16/18	80.0	32.9	26/27	71.7	33.6	*0.714*
Vitality	16/18	45.6	18.2	26/27	49.8	21.4	*0.654*
Mental health	16/18	64.3	13.6	26/27	61.5	15.0	*0.608*
Social functioning	16/18	72.6	26.3	26/27	73.1	21.7	*0.550*
Bodily pain	16/18	72.6	36.3	26/27	73.4	27.5	*0.143*
General health	16/18	59.5	22.4	26/27	53.1	24.5	*0.148*
ISP-25							
Global self-esteem	14/18	3.8	1.3	24/27	4.3	1.1	*0.419*
Physical self-worth	14/18	3.2	1.1	26/27	3.5	1.3	*0.129*
Physical condition	14/18	2.6	1.3	26/27	2.4	1.1	*0.461*
Sport competence	14/18	2.2	1.2	26/27	2.6	1.2	*0.228*
Attractive body	14/18	3.5	1	26/27	3.9	1	*0.250*
Physical strength	14/18	2.4	1.3	26/27	2.3	1.1	*0.866*

*N*: number of respondents; *n*: number of observations.

**Table 3:** Pregnancy outcomes after administration of I^131^ in subjects diagnosed with differentiated thyroid cancer before age of 25 years.

	Cumulative dose of I^131^	
	≤3.85 GBq (*N* = 18)	>3.85 GBq (*N* = 27)
Maternal age at pregnancy (yrs)	26.3 ± 5.1	27.3 ± 2.4
Interval between I^131^ and pregnancy (months)	69.7 ± 47.8	75.3 ± 64.8
Cumulative dose of I^131^ before pregnancy^†^	3.74 ± 0.08^†^	7.88 ± 1.3^†^
Intention to have children		
Patients already having children	6	15
Patients without children	12	12
Fertility problems after I^131^		
Pregnancy after intrauterine insemination	2	0
Pregnancy after FIV ICSI	1 (ongoing)	0
Dysovulation	0	1
Pregnancy outcomes		
Voluntary abortion	0	0
Miscarriage during the first trimester	1	5
Medical abortion	0	1
Live births	9	24
Birth term (weeks)	40 ± 1.2	38.7 ± 2.4
Birth weight (g)	3.256 ± 0.2	3.116 ± 0.6
Gender (male)	5	15
Number of patients having children	6	15
Number of children after I^131^	9	24

*N*: number of subjects. Data are presented as mean (±SD) or number (percentage).

^†^ *P* < 0.001.

## Abbreviations

DTC: differentiated thyroid cancer
HRQoL: health related quality of life
RAI: radioiodine ablation.
